# An epidemiological analysis of imported malaria in Shanghai during a COVID-19 outbreak

**DOI:** 10.1186/s12936-022-04273-9

**Published:** 2022-08-25

**Authors:** Min Zhu, Chengang Zhang, Yaoguang Zhang, Zhenyu Wang, Xiaojiang Ma, Simin Dai, Jian Chen

**Affiliations:** Shanghai Municipal Center for Disease Control & Prevention, Shanghai, People’s Republic of China

**Keywords:** Malaria, Imported case, Epidemiology, COVID-19, Shanghai

## Abstract

**Background:**

The goal of this study was to understand the epidemiological characteristics of imported malaria in Shanghai specifically during the epidemic period of novel corona-virus pneumonia (COVID-19), to provide a reference for preventing the transmission of imported malaria after this disease had been previously eliminated.

**Methods:**

The data of malaria cases reported in Shanghai from 2020 to 2021 were obtained from the China Information System for Disease Control and Prevention (CISDCP) and the Information System for Parasitic Disease Control and Prevention (ISPDCP). The characteristics of demographic and epidemiological distribution, travel-related information, diagnosis information, regions of infection acquisition and disposal information of epidemic situation were analysed with descriptive statistics.

**Results:**

A total of 112 cases of malaria were reported in Shanghai from January 2020 to December 2021. There were 18 cases and 94 cases in 2020 and 2021, respectively, reaching the lowest and highest levels in the past 10 years. The incidence of malaria associated with seasons had an increasing trend (χ^2^ = 81.143, P < 0.05). These cases included *Plasmodium falciparum* (97, 86.61%), *Plasmodium vivax* (4, 3.57%), *Plasmodium ovale* (8, 7.14%) and *Plasmodium malariae* (3, 2.68%). The median age of patients with malaria was 38.0 years, the majority of these individuals were males (109, 97.32%), and most of them were labour personnel (93, 83.04%). Of the reported cases, 8 of these individuals (7.14%) reported experiencing malaria symptoms before their arrival in China after their stay overseas; 97 of these individuals (86.61%) reported experiencing symptoms within 14 days after their initial arrival from overseas; 15 of these individuals (13.39%) were diagnosed with ‘severe malaria’; and 4 of these individuals (3.57%) were also diagnosed with COVID-19. All cases were imported from Africa, and there were no indigenous cases and deaths.

**Conclusion:**

Due to the impact of COVID-19, the number of imported malaria cases in Shanghai had greatly increased; however, prevention and control measures for imported malaria could be implemented to prevent re-transmission of this condition. Considering that the number of individuals returning from overseas labour is likely to increase in the next few years, it is necessary to strengthen the surveillance of imported malaria and to review the protocol for potential epidemic situations. Together, these measures could support the maintation of free-malaria status in Shanghai.

## Background

Malaria is one of the most important and prevalent infectious diseases in tropical and subtropical climates. Malaria continues to be a major public health issue. Malaria has previously impacted the population by seriously endangering human health, life and safety in China. Malaria had a peak incidence of 2961 per 100,000 individuals in the population in the year 1970. After decades of control efforts, the total population at risk in China has been shrinking; only some areas of the Yunnan Province reported indigenous cases of *Plasmodium falciparum* up to the year 2010 [1].

Shanghai is located in the Yangtze River Delta in eastern China, at the mouth of the Yangtze River near the East China Sea, and is connected with Jiangsu and Zhejiang provinces in the north and west. Shanghai has a northern subtropical monsoon climate with four distinct seasons. The annual average temperature is 16.0 °C, and January is the coldest month, with an average temperature of 4.9 °C. Typically, July is the hottest month, with an average temperature of 30.9 °C. The annual average rainfall is 1158 mm, and about 70% of it is concentrated in the flood season from May to September. According to mosquito monitoring data, *Culex pallens* has been the main mosquito species in Shanghai for nearly 10 years, accounting for about 80% of all mosquitoes, followed by *Aedes albopictus* (12%), *Culex tritaeniorhynchus* (5%,) *Armigeres subalbatus* (2%), and *Anopheles sinensis*, which is the least populous species and the only known vector of malaria, accounting for less than 1%. *Anopheles sinensis* is active from May to October, which is the highest transmission period of malaria. In Shanghai, *Plasmodium vivax* was once the predominant *Plasmodium* species of malaria, and the incidence rate was as high as more than 323 per ten thousand in 1962. After vigorous prevention and control, the incidence rate fell to less than one per ten thousand in 1984 [2].

In 2010, the Chinese government began launching National Action Plan of Malaria Elimination (NAPME) to achieve malaria-free status by 2020 [3]. There had been no indigenous malaria cases in Shanghai since 2010, and Shanghai was the first city to pass the provincial assessments of malaria elimination in 2016 [4]. By 2020, China was malaria-free, and by maintaining zero indigenous malaria cases for four consecutive years, China obtained malaria-free certification from the WHO in June 2021 [5].

However, thousands of imported malaria cases were still reported in China every year [6–8], and approximately 50 cases were reported every year in Shanghai [9–11]. In addition, *Anopheles* still exists as a vector for malaria transmission [12]. Considering this, the reestablishment of imported malaria transmission remains a potential risk. Therefore, malaria cases should be discovered, reported, and treated in a timely manner to clear the source of infection as early as possible and to carry out investigation and assessment quickly. For the epidemic spots with transmission risk, disposal measures mainly focusing on the control of mosquito vectors are undertaken to effectively prevent transmission. Since 2017, the “1 − 2 − 3 + 1” working requirement for malaria epidemic response has been implemented in Shanghai, which means that the reported case is conformed within one day, epidemiological investigation is completed within 2 days, epidemic foci are classified and disposed of within three days, and the evaluation of transmission blocking is implemented within one month. Therefore, the imported malaria cases are quickly detected, and their foci are promptly disposed of to prevent successful transmission. As a result, a malaria-free status has been maintained in Shanghai.

At the end of December 2019, COVID-19 pneumonia cases appeared in Wuhan, Hubei Province, and other regions in China [13, 14]. These gradually spread and displayed a global epidemic trend [15]. Individuals returning from malaria-endemic areas, such as Africa and Southeast Asia, were at risk of a double infection with COVID-19 and malaria. This resulted in new challenges to the control of imported malaria [16]. To prevent the re-transmission of imported malaria during the COVID-19 epidemic situation, an epidemiological analysis was conducted on data regarding imported malaria during the period of a COVID-19 outbreak from 2020 to 2021 in the city of Shanghai.

## Methods

### Case surveillance data

Malaria cases were reported through the China Information System for Disease Control and Prevention (CISDCP) within 24 h after cases were found by medical institutions at all levels. Then, the local county Center for Disease Control and Prevention (CDC) confirmed the case information within 24 h. Laboratory confirmation of *Plasmodium* was done using microscopic examination of blood smears, which was completed within 2 days. COVID-19, as a new infectious disease, has also been included in CISDCP; its reporting and review should be within 2 h.

A retrospective analysis using malaria data from the CISDCP from 2020 to 2021 was carried out. The basic malaria case information included date of birth, gender, occupation, usual residence and details of malaria illness such as date of onset of symptoms, date of diagnosis, date of treatment, method of diagnosis, severity, and information surrounding COVID-19 infection.

### Epidemiological data

Additional epidemiological information was obtained from the Information System for Parasitic Disease Control and Prevention (ISPDCP). After medical institutions submitted the malaria case report to the national CISDCP system, the local county CDC interviewed malaria patients and reported the epidemiological information to ISPDCP within 2 days. A national case investigation form was used to collect additional epidemiological information, including details on travel history such as date of arrival to China, countries visited, purposes of travel and time of malaria onset abroad. Based on this information, county-level CDC staff determined the type of infection and risk of transmission. According to the Technical Scheme of Prevention to Imported Malaria Re-transmission, the treatment measures for different transmission risks were implemented within three days to control transmission. Then, the effect assessment was carried out within one month, and the disposal measures could be terminated when there was no transmission vector and no infectious source in the epidemic focus, such as when the case was cured or died.

### Overseas labour returnee data

Ten years of data from individuals dispatched to overseas labour were collected from Shanghai Statistical Yearbooks and the National Bureau of Statistics. The number of returnees to Shanghai each year was calculated using the following formula:

$${\text{R}}_{{{\text{si}}}} = \left( {{\text{D}}_{{{\text{ni}}}} + {\text{O}}_{{{\text{n}}({\text{i}} - {\text{1}})}} - {\text{O}}_{{{\text{ni}}}} } \right) \times {\text{D}}_{{{\text{si}}}} \div {\text{D}}_{{{\text{ni}}}}$$

In the above equation, R_si_ refers to the returnees of Shanghai in each year, D_ni_ refers to the individuals of China dispatched overseas each year, O_ni_ refers to the individuals of China who remained dispatched overseas at the end of each year and D_si_ refers to the individuals of Shanghai dispatched overseas in each year.

### Definition of malaria cases and epidemic foci

Malaria cases with laboratory-confirmed *Plasmodium* infections that were reported to the CISDCP in 2020 and 2021 were included in the current study. An imported malaria case was defined as a malaria case with a travel history in overseas malaria-endemic areas before onset, or with clear overseas infection epidemiology and no evidence of local transmission. An indigenous case was defined as a malaria infection from the local transmission with no travel history of overseas malaria-endemic areas and locally acquired transmission cannot be disproven [17]. 

Epidemic foci of malaria were defined as places where malaria cases are located.

### Statistical analysis

Descriptive statistics were completed for all important variables. The distributions of counting data were assessed with Chi-square analysis. Trend regression analysis was used to analyse the seasonal distribution of malaria cases and the yearly distribution of overseas returnees’ labour. Statistical significance was set at 5%. SPSS Statistics Trial version (IBM Corporation) was used in this study.

## Results

### General situation of malaria case surveillance

A total of 112 malaria cases were reported in Shanghai in 2020 and 2021, with18 cases in 2020 and 94 cases in 2021, reaching the lowest and highest levels since 2010. Among these, 111(99.10%) of these cases were Chinese, with 3 (2.68%) of these registered in Shanghai and 108 (96.43%) of these registered in other provinces. Only one case was reported as a foreign national. All cases were found to be imported malaria without any indigenous cases, and no deaths occurred from 2020 to 2021.

### ***Plasmodium*** species

All of these 112 malaria cases were confirmed by the laboratory, including 97 cases of *P. falciparum* (86.61%), 4 cases of *P. vivax* (3.57%), 8 cases of *Plasmodium ovale* (7.14%) and 3 cases of *Plasmodium malariae* (2.68%). The cases of *P. falciparum* accounted for 83.% (15/18) in 2020 and 87.23% (82/94) in 2021 (χ^2^ = 0.005, *P* > 0.05).

### Characteristics of demographic and epidemiological distribution

**Fig. 1:**
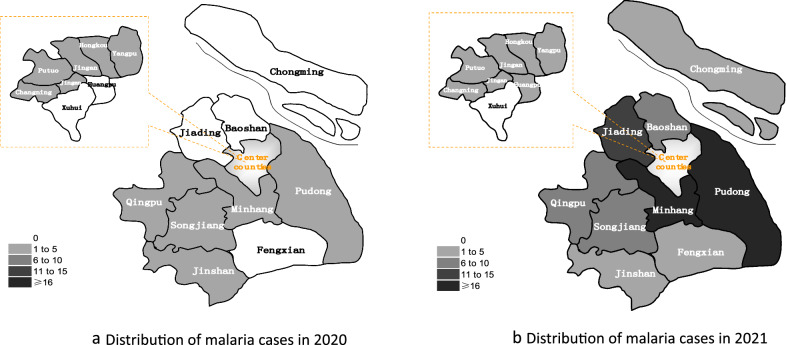
Distribution of malaria cases reported in Shanghai, 2020–2021

**Fig. 2:**
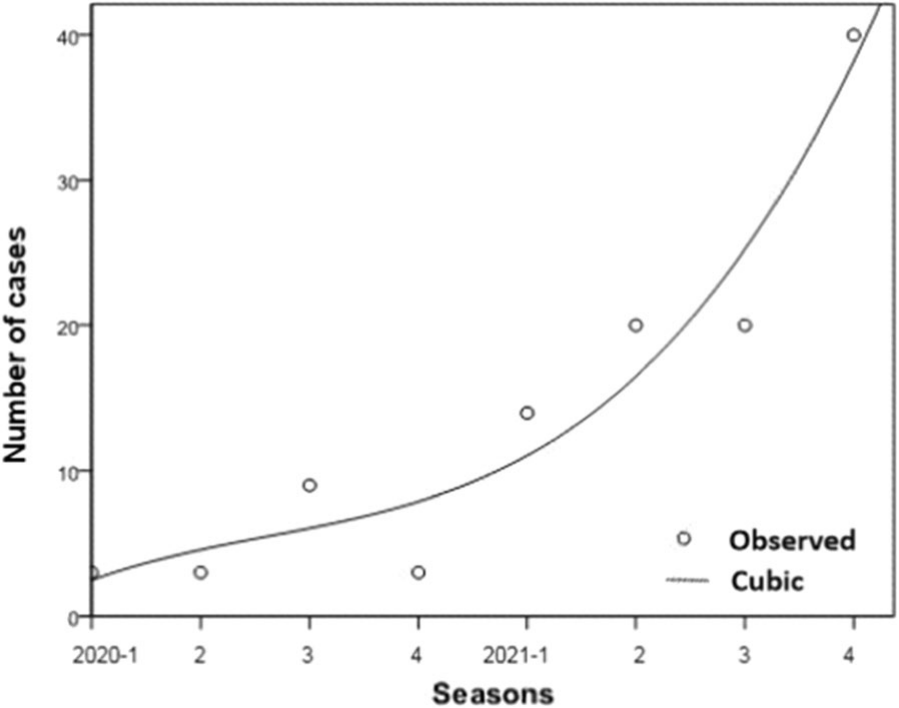
Seasonal trend of malaria cases from 2020 to 2021 in Shanghai

The median age of these cases was 38.0 years (range 21–65), the majority of these individuals were males (109, 97.32%), and most of them were labour personnel (83.04% (93)) (Table 1). All cases were reported from 15 districts of Shanghai, and 57.14% (64) of cases were reported in Pudong, Minhang, Jiading and Songjiang districts (Fig. 1). The average number of cases counted by quarter was 14 (range 3–40). The malaria cases numbers of quarter from 2020 to 2021 were respectively 3, 3, 9, 3, 14, 20, 20, 40, which presented an increasing trend with cubic index (*F* = 16.104, *P* < 0.05, R^2^_cubic_ = 0.924) (Fig. 2).

**Table 1:** Demographic characteristics of malaria cases in Shanghai, 2020–2021

Variable (n = 112)	Categories	2020		2021		Total	
		No.	%	No.	%	No.	%
Sex	Male	18	100.00	91	96.81	109	97.32
	Female	0	0.00	3	3.19	3	2.68
Age	20–29	3	16.67	17	18.09	20	17.86
	30–39	6	33.33	31	32.98	37	33.04
	40–49	4	22.22	27	28.72	31	27.68
	50–59	4	22.22	18	19.15	22	19.64
	60–69	1	5.56	1	1.06	2	1.79
Occupation	Labour personnel	10	55.56	83	88.30	93	83.04
	Business personnel	7	38.89	11	11.70	18	16.07
	Student	1	5.56	0	0.00	1	0.89

### Characteristics of travel-related information

The study results showed that the travelling and health information of 112 patients with malaria was captured. Of these patients, 8 (7.14%) had malaria symptoms before their arrival in China after staying overseas; 100 (89.28%) had symptoms within 30 days after their arrival from staying overseas, with 97 (86.61%) and 3 (2.68%) within 0–14 days and 15–21 days, respectively; and the other 4 (3.57%) cases had symptoms during 3–12 months after their arrival from staying overseas, including 3 cases of *P. ovale* and one case of *P. vivax.* Ninety-five (97.94%) *P. falciparum* cases and 10 (66.67%) other *Plasmodium* cases had malaria symptoms within 14 days of their return from staying overseas (χ^2^ = 16.673, P < 0.05) (Table 2).

**Table 2:** Interval days from date of arrival to date of symptom onset of malaria reported in Shanghai, 2020–2021

Interval (days) (n = 112)	*P. falciparum*	*%*	*P. vivax*	*%*	*P. ovale*	*%*	*P. malariae*	%	Total	%
< 0	8	8.25	0	0.00	0	0.00	0	0.00	8	7.14
0–14^a^	87	89.69	3	75.00	5	62.50	2	66.67	97	86.61
15–21^b^	2	2.06	0	0.00	0	0.00	1	33.33	3	2.68
91–180	0	0.00	0	0.00	2	25.00	0	0.00	2	1.79
181–360	0	0.00	1	25.00	1	12.50	0	0.00	2	1.79

^a^The centralized isolation period of COVID-19 prevention and control

^b^The health monitoring period of COVID-19 prevention and control

Of all cases, 65 cases (58.04%) had a history of malaria while staying overseas, including 52 cases of *P. falciparum*, 4 cases of *P. vivax*, 7 cases of *P. ovale* and 2 cases of *P. malariae*. In addition, 40 (35.71%) cases carried anti-malarial drugs to control the onset of fever at any time, and 25 (22.32%) cases took anti-malarial drugs to control fever while feeling unwell.

### Characteristics of diagnosis and treatment information

All 112 malaria cases were reported within 24 h after being diagnosed by 30 medical institutions, including 7 provincial-level and 23 county-level institutions, with 19 (16.96%) and 93 (83.04%) cases reported, respectively. The median number of days of interval from onset of malaria symptoms to diagnosis was 1 day (range 0–10 days). As many as 91 (81.25%) malaria cases were diagnosed within 3 days, with 13 (72.22%) and 78 (82.98%) in 2020 and 2021, respectively (χ^2^ = 0.55, *P* > 0.05). These were recorded as 13 (68.4%) and 78 (83.9%) in provincial-level and county-level medical institutions, respectively (χ^2^ = 1.562, *P* > 0.05).

Among these 112 malaria cases, 15 (13.39%) cases were diagnosed as severe malaria, all of which were *P. falciparum.* Of these, 5 individuals (30%) had a history of malaria infection during their travels of staying overseas while 60 (61.85%) out of 97 individuals reported having non-severe malaria (χ^2^ = 4.340, *P* < 0.05).

Four (3.57%) of these cases were confirmed to have COVID-19 as well. All of these cases were *P. falciparum* with 1 case of severe malaria and 2 cases with a history of malaria. These 4 cases were all Chinese men aged 40, 45, 46 and 54 years who returned from Ghana, Mali, Guinea and Ghana, respectively. These individuals were diagnosed with malaria at 13, 7, 3 and 13 days after their arrival from staying overseas and were diagnosed with COVID-19 at 5, 4, 14 and 22 days.

All malaria cases were treated with artemisinin, following the industry standard for the use of anti-malarial drugs formulated by the People’s Republic of China’s Ministry of Health. For severe malaria, artesunate injection was used, and artemisinin piperaquine oral tablets were taken for non-severe malaria, while for malaria cases of *P. vivax* and *P. ovale*, primaquine phosphate was added to prevent relapse. All cases were cured without deaths, and no cases of drug-resistant malaria were found.

### Regions of infection acquisition

All of these 112 malaria cases were imported with country information available on the location of suspected infection acquisition, and all imported cases of infections were acquired from 22 countries among those travelling to Africa. Malaria cases imported from Guinea, Nigeria, the Democratic Republic of Congo, Ethiopia and Côte d’Ivoire accounted for 70.54% (79) of total imported cases (Table 3).

**Table 3:** Regions of infection acquisition of imported malaria in Shanghai, 2020–2021

Country of acquisition (n = 112)	*P. falciparum*	*P. vivax*	*P. ovale*	*P. malariae*	Total
Guinea	32		5	1	38
Nigeria	13		2	1	16
Congo DR	11				11
Ethiopia	3	4			7
Côte d’Ivoire	6		1		7
Congo	3			1	4
Ghana	4				4
Benin	3				3
Mali	3				3
Sierra Leone	3				3
Zambia	3				3
Togo	2				2
Cameroon	2				2
Other African countries (9)	9				9

### Disposal information of epidemic situation

Epidemiological investigation, disposal and evaluation were carried out on all cases, and 119 (97.32%) of these case investigations were completed within 2 days. A total of 84 epidemic foci were identified with no possibility of transmission. A total of 157 colleagues of known malaria patients were screened for malaria parasites, and no new infected individuals were found. Risk assessments after one month showed that all cases were cured with no reported deaths and no re-transmission.

### Returnee information

**Fig. 3:**
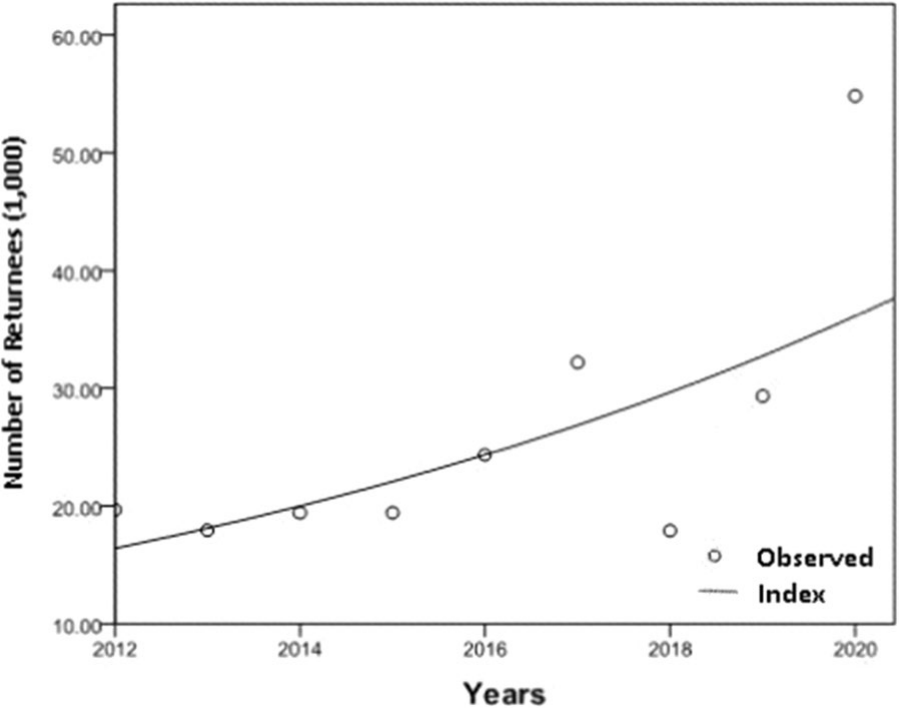
Returnees trend of overseas labour in Shanghai from 2012 to 2020

**Fig. 4:**
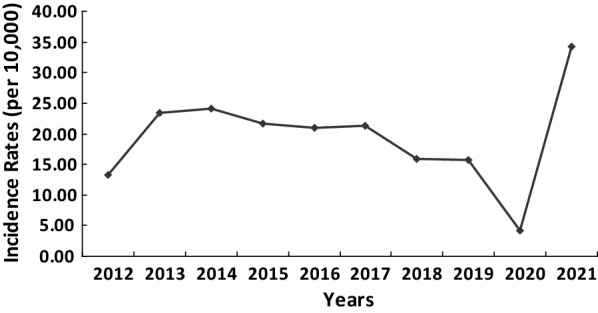
Malaria incidence rates of returnees from overseas labour in Shanghai from 2012 to 2021

Complete data regarding national overseas labour from 2011 to 2020 were obtained, and nearly ten years of data from returnees from overseas labour in Shanghai were calculated out with a yearly average of 26,120 people (range: 17,906–54,820), which presented an index increasing trend (*F* = 7.808, *P* < 0.05, R^2^_index_ = 0.527) (Fig. 3). In the past 10 years, the malaria incidence per 10,000 returnees from overseas labour in Shanghai rose from 4.28 to 34.29, reaching the lowest and highest incidence rates in 2020 and 2021, respectively (Fig. 4).

## Discussion

COVID-19 has a wide epidemic range and a strong transmission capacity. It is mainly transmitted through contact with respiratory tract secretions and droplets [18]. Social isolation and disinfection are the main measures of prevention and control.

On January 30, 2020, the WHO announced that COVID-19 would be listed as a ‘public health emergency of international concern’ [19]. At this time, many countries and regions around the world restricted entry and exit, airlines reduced or grounded flights and the number of people entering China dropped sharply. This also led to the national malaria epidemic falling to the lowest level in history [20]. This analysis also found that the number of malaria cases reported in Shanghai in 2020 was significantly lower than that in previous years [8–10], reaching the lowest level since the elimination of malaria. With the phased victory in the prevention and control of COVID-19 in China, the domestic epidemic had been effectively controlled [21], but the overseas epidemic was spreading rapidly. This showed a global pandemic situation [22] that led to an increasing number of individuals returning from overseas. As a major city of entry, Shanghai has undertaken nearly one-third of the country’s inbound and outbound flights [23]. According to the requirements for the prevention and control of the COVID-19 virus, inbound personnel should be isolated locally in the city of entry for 14 days before going to their final destination [24]. This resulted in a rapid increase in the number of imported malaria cases.

This paper analysed the number of malaria cases reported in Shanghai in 2021 and found that this had increased to the highest level over recent years [9–11]. More than 96% of imported malaria cases reported a registered residence in other provinces of China and more than 86% of these cases had malaria symptoms during the centralized isolation period of COVID-19.

This paper analysed the results of an increasing trend of imported malaria cases and individuals returning from labour overseas. When considering the improvement of the global epidemic situation of COVID-19, it cannot be ruled out that a large number of people returned home from overseas after spending time in malaria-endemic areas, leading to a subsequent outbreak of imported malaria [25]. It is necessary to strengthen the joint prevention and control mechanisms of multiple departments to improve the effective emergency response of increasing cases of imported malaria. This is just as important as the prevention and control of COVID-19 for those individuals returning from overseas where they visited malaria-endemic areas. Malaria monitoring, screening and risk assessment should be carried out to identify cases of imported malaria and prevent transmission to others.

According to the World Health Organization’s World Malaria Report, 241 million cases of malaria cases and 627,000 malaria-related deaths were estimated in 2020. This displayed an increase of 14 million and 69,000, as compared with data from 2019. Africa accounted for approximately 95% of these cases and 96% of these deaths globally [26]. The cases analysed in this paper were all imported from Africa. Of these patients, nearly 60% had a history of malaria that had been diagnosed while staying abroad, and more than 20% took anti-malarial drugs to control a fever, although they did not fully complete malaria treatment [27]. This easily led to recrudescence or the emergence of anti-malarial drug resistance. Therefore, it is necessary to focus on the publicity of malaria prevention, control knowledge, monitoring and screening for high-risk personnel that are both entering and leaving malaria-endemic areas. This can help achieve the goals of early detection, early diagnosis and early treatment.

It is well known that the incubation period of malaria is generally 11 to 40 days [28], while the incubation period is 1–14 days for COVID-19 [29], with an overlap period in epidemiology and infection incubation among both of these conditions.

This paper demonstrated that approximately 94% of imported malaria cases were identified within 14 days of the centralized isolation period for COVID-19 prevention and control. Therefore, this management measure for inbound personnel could not only effectively prevent the transmission of imported COVID-19 in the local area but also provide a guarantee for the early detection, diagnosis and treatment of imported malaria. This study found that the median number of days from onset of malaria symptoms to diagnosis was only 1 according to data from 2020 to 2021. This was greatly shortened compared with the 4.4 days that was reported in previous years [9]. In addition, this type of malaria had similar clinical manifestations to COVID-19 mainly characterized by fever, which may have easily led to missed diagnosis or incorrect diagnosis of malaria. In addition, the dual infection of *Plasmodium* and COVID-19 may exist in the entry personnel from overseas who have visited malaria-endemic areas. This paper found that 4 cases were diagnosed with both *Plasmodium* and COVID-19 simultaneously. Therefore, medical staff should review the differential diagnosis for a case in real time and be mindful of personal protection protocols to prevent COVID-19 infection [30]. In addition, according to the requirements of prevention and control for COVID-19, the diagnosis and treatment for patients with fever should be completed in designated hospitals. These were at least county-level medical institutions in every district. The results of this study showed that more than 83% malaria cases were diagnosed in county-level medical institutions in 2020 and 2021. In previous years, nearly 80% of malaria cases were diagnosed in provincial-level medical institutions [10].

During the period of prevention and control of COVID-19, it is necessary to further strengthen the professional training for malaria detection, diagnosis and treatment in county-level medical institutions. This will help prevent missed diagnosis and incorrect diagnosis of malaria, and will hopefully avoid malaria deaths caused by delayed diagnosis or irregular treatment.

## Conclusion

During the COVID-19 epidemic, due to the implementation of management measures to restrict personnel mobility, there was a great change in the reported incidence of imported malaria. It is possible to avoid this potential new epidemic with measures to control imported malaria that could be carried out regularly. These actions could effectively prevent re-transmission of imported malaria. In the future, officials should continue to strengthen the monitoring of malaria and other communicable diseases to be prepared for an emergency response to imported malaria and other conditions that may need to be eliminated.

## Data Availability

Data supporting the conclusions of this article are included within the article. The datasets used and/or analysed during the present study are available from the corresponding author upon reasonable request.

## References

[1] Tang LH, Xu LQ, Chen YD (2012). Parasitic diseases control and research in China.

[2] Cai L, Zhu M, Wu HY (2019). Data compilation of malaria elimination in Shanghai.

[3] Ministry of Health of the People’s Republic of China. Action Plan of China Malaria Elimination (2010–2020) (**in Chinese**). http://www.nhc.gov.cn/jkj/s5873/201005/f84f1c4b0f32420990d23b65a88e2d87.shtml

[4] Zhu M, Cai L, Wu HY, Wang ZY, Zhang YG, Jiang L (2018). Mid-term assessment report of malaria elimination action plan in Shanghai. Chin Trop Med J.

[5] Feng J, Zhang L, Xia ZG, Zhou SS, Xiao N (2021). Malaria-free certification in China: achievements and lessons learned from the national malaria elimination programme. Zoonoses.

[6] Zhang L, Feng J, Zhang SS, Xia ZG, Zhou SS (2018). The progress of national malaria elimination and epidemiology characteristic of malaria in China in 2017. Chin J Parasit Dis.

[7] Zhang L, Feng J, Zhang SS, Xia ZG, Zhou SS (2019). Epidemiological characteristic of malaria and the progress towards its elimination in China in 2018. Chin J Parasit Dis.

[8] Zhang L, Feng J, Xia ZG, Zhou SS (2020). Epidemiological characteristic of malaria and the progress on its elimination in China in 2019. Chin J Parasit Dis.

[9] Zhu M, Cai L, Zhang CG, Zhang YG, Wang ZY (2019). Epidemiological analysis of malaria cases reported in Shanghai from 2010 to 2017. Chin J Parasit Dis.

[10] Zhu M, Wu HY, Zhang CG, Zhang YG, Wang ZY, Ma XJ (2021). Epidemiological analysis of imported malaria reported in Shanghai, 2016–2019. Chin Trop Med J.

[11] Dai SM, Zhu M, Wu HY, Zhang YG, Wang ZY, Zhang CG (2021). From malaria elimination to post-elimination: a 10-year surveillance data study in Shanghai. Malar J.

[12] Zhu M, Cai L (2014). Malaria surveillance in Shanghai from 2005 to 2012. Chin J Schisto Control.

[13] Zhu N, Zhang D, Wang W, Li X, Yang B, Song J (2020). A novel corona-virus from patients with puenumonia in China, 2019. N Engl J Med.

[14] Epidemiology Working Group for NCIP Epidemic Response, Chinese Center for Disease Control and Prevention (2020). The epidemiological characteristics of an outbreak of 2019 novel corona-virus disease (COVID-19) in China. Chin J Epidemiol.

[15] Wang C, Horby PW, Haden FG, Gao GF (2020). A novel corona-virus outbreak of global health concern. Lancet.

[16] Zhu GD, Cao J (2020). Challenges and countermeasures on Chinese malaria elimination programme during the corona-virus disease 2019 (COVID-19) out break. Chin J Schisto Control.

[17] Chinese Centre for Disease Control and Prevention. Technical scheme of China malaria elimination, 2011 edition. in Chinese. https://www.chinacdc.cn/did1/crbzt/jscb/nj/njzyzl/lgjbfk/lgfkfa/201507/P020150715347374233381.pdf.

[18] Li Q, Guan X, Wu P, Wang X, Zhou L, Tong Y (2020). Early transmission dynamics in Wuhan, China, of novel corona-virus-infected pneumonia. N Engl J Med.

[19] WHO. Statement on the second meeting of the International Health Regulation(2005) Emergency Committee regarding the outbreak of novel corona-virus (2019-nCov). Geneva: World Health Organization. 2020. https://www.who.int/news-room/detail/30-01-2020-statement-on-the-second-meeting-of-the-international-healthregulation-(2005)-emergency-committee-regarding-the-outbreak-of-novel-coronavirus(2019-nCov).

[20] Zhang L, Feng J, Tu H, Yin JH, Xia ZG (2021). Malaria epidemiology in China in 2020. Chin J Parasitol Parasit Dis.

[21] Di YN, Gao TD (2020). The Chinese people are making contributions to all mankind: a summary of overseas views on combating epidemic. Healthy China Observ.

[22] Mahase E (2020). Covid-19: WHO declares pandemic because of “alarming levels” of spread, severity, and inaction. BMJ.

[23] Chen SS, Ma CC, Xu HY. How much is the input pressure of Shanghai external defense? The data tell you the truth (**in Chinese**). https://www.yicai.com/news/101350912.html.

[24] Comprehensive group of the State Council on joint prevention and control mechanism for novel corona-virus pneumonia. Novel corona-virus pneumonia prevention and control program, 8th edition (**in Chinese**).

[25] Zhang J. Analysis of imported malaria cases in Nanning city. Chin Trop Med J. 2013;1415–6.

[26] WHO (2021). World malaria report 2021.

[27] Gao Q, Tang LH, Fu CL, Yu XB, Guan YY, Zhou SS, et al. WS/T 485-2016 Technical regulations for application of antimalarials. Health and Family Planning Commission of the People’s Republic of China. 2015 (**in Chinese**).

[28] Tang LH, Gao Q, Yu XB, Guan YY, Zhou SS, Zhou XN, Xia ZG. WS 259-2015 diagnosis of malaria. Health and Family Planning Commission of the People’s Republic of China, 2015 (**in Chinese**).

[29] General Office of the State Health Commission of the People’s Republic of China. Diagnosis and treatment of novel corona-virus pneumonia (trial version 9) (**in Chinese**). 202203/b74ade1ba4494583805a3d2e40093d88.shtml.

[30] National Health Commission. Notice of the general office of the National Health Commission on strengthening the management of fever clinics in key hospitals of important areas and the prevention and control of infection in medical institutions. /s7659/202002/485aac6af5d54788a05b3bcea5a22e34.shtml.

